# Comparative Study of Hot Pressing and Spark Plasma Sintering on the Phase Transformation, Microstructure, and Properties of Si_3_N_4_ Ceramics with YF_3_-MgSiN_2_ Additives

**DOI:** 10.3390/ma19153250

**Published:** 2026-08-01

**Authors:** Zihan Guo, Xiaoan Lv, Qing Qin, Xiaona Ren, Changchun Ge

**Affiliations:** 1 Institute of Powder Metallurgy and Advanced Ceramics (IPMAC), School of Materials Science and Engineering, University of Science and Technology Beijing (USTB), Beijing 100049, China; 18633659036@163.com (Z.G.); lvxiaoan29@126.com (X.L.); qinqing20@mails.ucas.ac.cn (Q.Q.); 2 School of Chemistry and Chemical Engineering, Hebei Minzu Normal University, Chengde 067000, China

**Keywords:** Si_3_N_4_, sintering process, mechanical properties, thermal conductivity, microstructure

## Abstract

Si_3_N_4_ ceramics with a YF_3_-MgSiN_2_ binary additive system were fabricated by hot pressing (HP) and spark plasma sintering (SPS) at 1500–1700 °C, followed by annealing at 1850 °C for 6 h. The effects of sintering route and temperature on phase transformation, microstructure evolution, thermal conductivity, and mechanical properties were systematically investigated. SPS significantly accelerated the α→β phase transformation compared with HP, and the β-Si_3_N_4_ content in SPS samples exceeded 94% at 1600 °C. After annealing, all samples were completely transformed into β-Si_3_N_4_, accompanied by obvious grain growth. Thermal conductivity was closely related to both grain size and relative density. Grain growth reduced grain-boundary phonon scattering, whereas density loss and residual porosity deteriorated heat transport. The mechanical properties were jointly governed by β-Si_3_N_4_ content, grain morphology, and porosity. Elongated β-Si_3_N_4_ grains promoted crack deflection and crack bridging, thereby improving fracture toughness, while excessive porosity reduced flexural strength. These results demonstrate that optimizing the balance between phase transformation, grain growth, and densification is essential for tailoring the thermal and mechanical performance of Si_3_N_4_ ceramics containing non-oxide sintering additives.

## 1. Introduction

Silicon nitride (Si_3_N_4_) ceramics are among the most important structural ceramics due to their exceptional combination of high strength, high hardness, excellent wear resistance, superior chemical stability, and low thermal expansion coefficient [1,2,3]. Furthermore, their outstanding high-temperature mechanical performance, thermal shock resistance, and electrical insulation properties make them attractive for a wide range of advanced engineering applications. Consequently, Si_3_N_4_ ceramics have been extensively utilized in mechanical, aerospace, electronic packaging, energy, and chemical industries, with promising applications in bearings, mechanical seals, turbine components, high-temperature structural parts, and electronic substrates [4,5,6].

Owing to the strong covalent bonding and low self-diffusion coefficient of Si_3_N_4_, densification through solid-state sintering is difficult to achieve because of the limited sintering driving force. Consequently, liquid-phase sintering with the aid of sintering additives is generally adopted for the fabrication of dense Si_3_N_4_ ceramics. The incorporation of suitable sintering additives plays a pivotal role in promoting densification. During the sintering process, these additives react with the native silica layer on the powder surface to generate a transient liquid phase, thereby enhancing particle wetting, facilitating dissolution–precipitation transport, and accelerating the α-to-β phase transformation of Si_3_N_4_. These processes collectively contribute to the densification and microstructural development of the ceramics [7,8,9].

Conventional sintering additives for Si_3_N_4_ ceramics are primarily oxide-based systems, such as Y_2_O_3_, Al_2_O_3_, and MgO, which effectively enhance sintering kinetics and facilitate densification [10,11,12]. However, these additives often result in the formation of amorphous grain-boundary phases that tend to soften and undergo viscous flow at elevated temperatures, thereby degrading the high-temperature strength, creep resistance, and thermal stability of the ceramics. Consequently, the application of Si_3_N_4_ ceramics under severe service conditions is restricted.

In recent years, non-oxide sintering additives have been extensively investigated as an alternative approach to further improve the performance of Si_3_N_4_ ceramics. Typical examples include fluorides [13,14], silicides [15,16], and hydrides [17,18]. Compared with conventional oxide additives, non-oxide additives can reduce the oxygen content, tailor grain-boundary chemistry, and improve high-temperature performance. Our research team has conducted systematic investigations into combinations of non-oxide sintering aids [19]. Among them, YF_3_ and MgSiN_2_ have been reported to effectively purify the Si_3_N_4_ lattice and decrease the oxygen concentration in the ceramics, leading to enhanced material properties [20]. MgSiN_2_ can effectively suppress the introduction of extrinsic oxygen, thereby increasing the N/O ratio in the liquid phase and promoting the growth of Si_3_N_4_ grains. However, an excessively high N/O ratio may significantly increase the viscosity of the liquid phase, limiting mass transport during liquid-phase sintering and consequently hindering densification. In addition, fluoride additives can react with SiO_2_ to form volatile SiF_4_, which further reduces the oxygen content of the system. Although this oxygen-scavenging effect is beneficial for grain growth and grain-boundary purification, the volatilization of SiF4 may also lead to density loss and increased residual porosity, thereby adversely affecting the densification of the ceramics [13,21]. Therefore, despite these advantages, the sintering processes involving non-oxide additives are generally more complicated than those associated with conventional oxide systems [14,16].

The sintering process not only determines the densification of the material but also directly influences the grain size, phase composition, grain-boundary structure, and ultimately the service performance of the ceramics. To achieve high-performance Si_3_N_4_ ceramics, appropriate sintering techniques are required to promote mass transport and particle rearrangement, thereby optimizing the microstructure [21,22,23].

At present, the most commonly employed sintering methods for Si_3_N_4_ ceramics include reaction bonding, hot pressing (HP), gas-pressure sintering (GPS), and spark plasma sintering (SPS). Among these techniques, HP and SPS are capable of producing ceramics with high density and excellent mechanical properties due to their enhanced densification efficiency. In contrast, GPS can effectively suppress the thermal decomposition of Si_3_N_4_ at elevated temperatures, enabling the fabrication of large and complex components with near-full density. In practical applications, different sintering techniques can be combined to take advantage of their respective merits, thereby providing an effective approach for tailoring the microstructure and properties of Si_3_N_4_ ceramics [13,20,23].

In this work, the sintering behavior of Si_3_N_4_ ceramics with a YF_3_-MgSiN_2_ binary additive system was systematically investigated. SPS and HP, followed by post-sintering annealing, were employed to fabricate the ceramics. The effects of sintering route and sintering temperature on densification behavior were comprehensively examined. In addition, the microstructural evolution and corresponding changes in the mechanical and thermal properties induced by annealing were systematically evaluated. By correlating processing conditions with microstructure and properties, this study aims to provide a deeper understanding of the sintering mechanisms associated with non-oxide additives and offer guidance for the development of high-performance Si_3_N_4_ ceramics.

## 2. Experimental

### 2.1. Material Preparation

**Figure 1:**
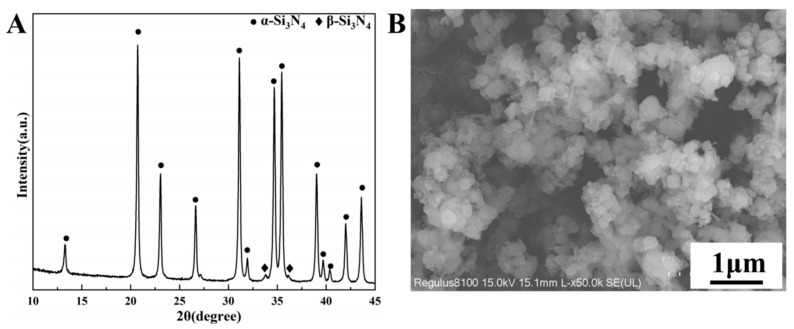
(**A**) XRD patterns of the α-Si_3_N_4_ powder. (**B**) SEM micrographs of the α-Si_3_N_4_ powder.

Raw materials included α-Si_3_N_4_ powder (SN-E10, D50 = 0.71 μm, oxygen content < 1.2 wt%, UBE Industries, Ltd., Tokyo, Japan). The XRD patterns and SEM micrographs of the α-Si_3_N_4_ powder are shown in Figure 1. YF_3_ (purity ≥ 99.9 wt%, Macklin Reagent Co., Ltd., Shanghai, China). MgSiN_2_ (Qilu Zhongke Institute of Photonics & Physics, Jinan, China).

The starting powders were mixed at a fixed molar ratio of Si_3_N_4_∶MgSiN_2_∶YF_3_ = 93∶5∶2. The powder mixture was ball-milled in ethanol with zirconia milling balls using a planetary mill for 4 h to ensure homogeneous mixing. The resulting slurry was subsequently vacuum-dried to minimize oxygen uptake and then sieved prior to sintering. The prepared powders were then consolidated either by hot pressing (ZT-40-21Y, Shanghai Chenhua Technology Co., Ltd., Shanghai, China) under vacuum at temperatures ranging from 1500 to 1700 °C and an axial pressure of 40 MPa for 1 h, or by spark plasma sintering (SPS-20T-10, Shanghai Chenhua Technology Co., Ltd., Shanghai, China) under the same temperature range and pressure for 5 min. Finally, the sintered specimens were annealed at 1850 °C for 6 h in a gas-pressure furnace (ZTQ-60-22, Shanghai Chenhua Technology Co., Ltd., Shanghai, China) under a 1 MPa N_2_ atmosphere to promote grain growth.

For convenience, the specimens were designated according to the sintering method and sintering temperature as HP-5, HP-6, HP-7, SPS-5, SPS-6, and SPS-7, respectively, with the detailed processing conditions listed in Table 1. The corresponding annealed specimens were labeled HP-5a, HP-6a, HP-7a, SPS-5a, SPS-6a, and SPS-7a, where the suffix “a” denotes the post-sintering annealing treatment.

### 2.2. Characterization

The samples were subjected to phase analysis using X-ray diffraction (XRD, STOE/2,3 KW, STOE, Darmstadt, Germany). The β-Si_3_N_4_ content was determined using the method proposed by Gazzara and Messier, expressed as follows [24]:

(1) Rβ = [Iβ(101)+Iβ(210)][Iα(102)+Iα(210)+Iβ(101)+Iβ(210)]×100%

The theoretical density of the samples was calculated based on their chemical composition. The bulk density was measured using the Archimedes method, and the relative density was determined from the ratio of the measured density to the theoretical density.

The microstructures of the powders and sintered ceramics were characterized by SEM (Regulus8100, Hitachi High-Tech Corporation, Tokyo, Japan). For powder characterization, the powders were mounted on conductive carbon tape and coated with gold prior to SEM observation.

For the ceramic samples, the surfaces were first polished and subsequently indented using a Vickers hardness tester (HVS-50Z-CCD, Suzhou Nanguang Electronic Technology Co., Ltd., Suzhou, China). The polished surfaces were then plasma etched in CF_4_ for 180 s to expose the grain boundaries and grain morphology. After gold coating, SEM observations were conducted. The average grain size was determined using Nano Measurer 1.2 software by analyzing no fewer than 300 grains for each specimen.

The sintered specimens were cut into the required dimensions for subsequent characterization. The flexural strength was measured by a three-point bending test (STD5000, Xiamen EAST Co., Ltd., Xiamen, China) using specimens with dimensions of 30 mm × 3 mm × 2 mm, a support span of 20 mm, and a crosshead speed of 0.5 mm/min. The reported values represent the average and standard deviation obtained from five specimens. The Vickers hardness and fracture toughness were determined by the indentation method using a hardness tester (HKV-50Z-CCD, Suzhou Nanguang Electronic Technology Co., Ltd., Suzhou, China) under a load of 98 N with a dwell time of 15 s. Five measurements were performed for each sample, and the average values together with the corresponding standard deviations were calculated. The fracture toughness (K*_IC_*) was estimated using the indentation fracture method according to the following equation. Young’s modulus of 300 GPa was used [25]:

(2) KIc = 0.016(EH)0.5(PC1.5)

The thermal conductivity was determined by the laser flash analysis (LFA) method using a laser flash apparatus (LFA 457 HyperFlash, NETZSCH Instruments GmbH, Selb, Germany). Specimens with dimensions of 10 mm × 10 mm × 2 mm were used for the measurements. In this study, the specific heat capacity of Si_3_N_4_ was assumed to be a constant value of 0.68 J·g^−1^·K^−1^. All property measurements were conducted at 25 °C.

## 3. Results and Discussions

### 3.1. Phase and Density

**Figure 2:**
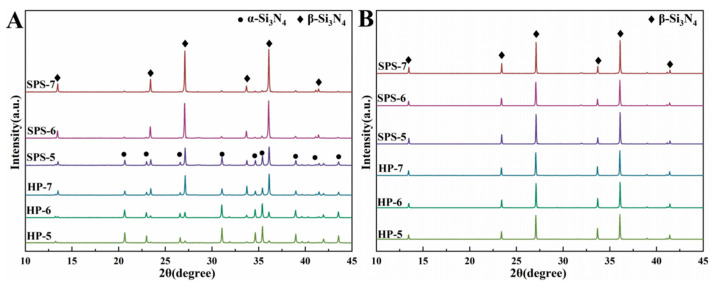
The XRD patterns of Si_3_N_4_ ceramics: (**A**) before annealing; (**B**) after annealing.

Figure 2 presents the XRD patterns of the Si_3_N_4_ ceramics. Figure 2A shows the XRD patterns of the as-sintered samples, whereas Figure 2B shows those of the annealed samples.

As shown in Figure 2A, HP-5 and HP-6 exhibit relatively strong α-Si_3_N_4_ diffraction peaks and weak β-Si_3_N_4_ peaks, indicating that α-Si_3_N_4_ remains the dominant crystalline phase and that only a limited fraction of α-Si_3_N_4_ has transformed into β-Si_3_N_4_. In contrast, the intensity of the β-Si_3_N_4_ diffraction peaks increases significantly in HP-7, suggesting that the α→β phase transformation is markedly accelerated during hot pressing at 1700 °C. Compared with HP, SPS exhibits a substantially higher phase transformation rate. Distinct β-Si_3_N_4_ diffraction peaks can already be observed in SPS-5, indicating that a considerable amount of α-Si_3_N_4_ has transformed into β-Si_3_N_4_ after SPS at 1500 °C for only 5 min. This behavior may be attributed to the unique heating mechanism of SPS, in which rapid Joule heating generated by the applied electric current can produce localized high-temperature regions within the powder compact. Such conditions facilitate the formation of the liquid phase and enhance the dissolution–precipitation process, thereby accelerating the α→β phase transformation. Consequently, the SPS samples contain a higher fraction of β-Si_3_N_4_ than the HP samples processed at the same nominal temperature.

As shown in Figure 2B, all diffraction peaks can be indexed to β-Si_3_N_4_. Furthermore, the diffraction peaks are sharp and intense for all samples, indicating a high degree of crystallinity. These results demonstrate that, regardless of the initial α/β phase ratio, all samples underwent a complete α→β phase transformation during the subsequent annealing treatment conducted under identical conditions, resulting in well-crystallized β-Si_3_N_4_ ceramics.

**Figure 3:**
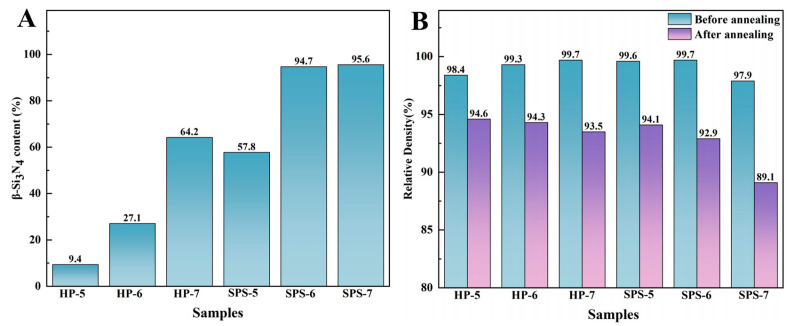
(**A**) β-Si_3_N_4_ content of Si_3_N_4_ ceramics. (**B**) Density of Si_3_N_4_ ceramics.

Figure 3A shows the β-Si_3_N_4_ phase fraction of the as-sintered samples. The β-Si_3_N_4_ content in HP-5 was only 9.4%, indicating that only a small fraction of α-Si_3_N_4_ underwent dissolution–precipitation and transformed into β-Si_3_N_4_ during hot pressing at 1500 °C. When the sintering temperature was increased to 1600 °C, the β-Si_3_N4 content in HP-6 increased to 27.1%, suggesting that the α→β phase transformation became more pronounced, although the transformation rate remained relatively slow. Upon further increasing the temperature to 1700 °C, the β-Si_3_N_4_ content in HP-7 reached 64.2%, exceeding 50%. This result indicates a substantial acceleration of the phase transformation process, with β-Si_3_N_4_ becoming the dominant crystalline phase. In contrast to the HP samples, the SPS samples exhibited significantly higher β-Si_3_N_4_ contents. In SPS-5, the β-Si_3_N_4_ fraction reached 57.8%, already becoming the major phase in the ceramic. With further increases in temperature, the β-Si_3_N_4_ contents in SPS-6 and SPS-7 increased to 94.7% and 95.6%, respectively. These results indicate that the α→β transformation proceeds rapidly during SPS at 1600 °C. The relatively small increase in β-Si_3_N_4_ content between SPS-6 and SPS-7 suggests that further temperature increases have a limited effect on phase transformation and that the holding time may become the primary factor governing the transformation extent under these conditions.

Figure 3B presents the relative densities of the samples before and after annealing. Prior to annealing, all samples exhibited relatively high densities. However, the relative density of HP-5 was only 98.4%, which is lower than 99%. Combined with its low β-Si_3_N_4_ content, this result suggests that the amount of liquid phase generated at 1500 °C was limited and that the liquid phase likely possessed a relatively high viscosity, resulting in insufficient particle wetting and incomplete densification [26]. In addition, SPS-7 exhibited a relative density of 97.9%, which may be associated with partial Si_3_N_4_ decomposition and volatilization of the liquid phase caused by localized overheating during SPS, thereby reducing the final density. After annealing, all samples exhibited a decrease in relative density. This reduction may be attributed to the reaction of F-containing species from the sintering additives with SiO_2_ and Si_3_N_4_, leading to the formation and volatilization of gaseous fluorine-containing compounds such as SiF_4_. Furthermore, evaporation of a small amount of intergranular phase may also contribute to the density loss [13,23]. Among all samples, SPS-6a and SPS-7a exhibited a more pronounced decrease in relative density, with SPS-7a showing the lowest value of only 89.1%. This behavior may be related to the earlier formation of β-Si_3_N_4_ during SPS. The elongated β-Si_3_N_4_ grains interlocked to form a rigid grain skeleton within the ceramic. During subsequent annealing and grain growth, the growth space available for these rod-like grains became increasingly constrained. Consequently, when part of the intergranular phase volatilized, the resulting pores were more difficult to eliminate or fill through grain rearrangement and mass transport, leading to a more significant reduction in density compared with the other samples.

### 3.2. Microstructure and Grain Diameter Distribution

**Figure 4:**
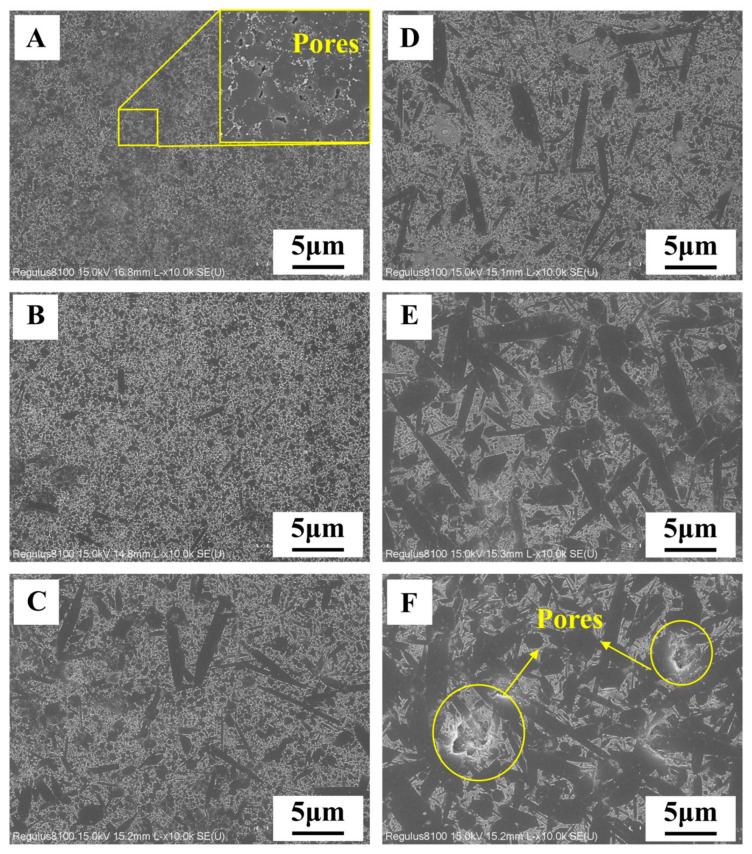
SEM images of the as-polished and plasma-etched surfaces of Si_3_N_4_ ceramics: (**A**) HP-5, (**B**) HP-6, (**C**) HP-7, (**D**) SPS-5, (**E**) SPS-6, (**F**) SPS-7.

Figure 4 presents SEM micrographs of the polished surfaces of the as-sintered samples. The microstructure of HP-5 is dominated by equiaxed α-Si_3_N_4_ grains. Higher-magnification observations reveal that the intergranular phase does not completely fill the spaces between adjacent grains, and small gaps can still be observed. This observation is consistent with the relatively lower density of HP-5 shown in Figure 3B. For HP-6, a limited number of elongated β-Si_3_N_4_ grains can be observed, although their size remains relatively small. In HP-7, a typical duplex microstructure consisting of elongated β-Si_3_N_4_ grains and equiaxed α-Si_3_N_4_ grains is clearly evident, indicating a significant progression of the α→β phase transformation during sintering. The microstructure of SPS-5 is similar to that of HP-7, exhibiting elongated β-Si_3_N_4_ grains surrounded by residual equiaxed α-Si_3_N_4_ grains. In SPS-6 and SPS-7, the amount of β-Si_3_N_4_ increases substantially, and the elongated grains become more fully developed with significantly larger sizes. The rod-like β-Si_3_N_4_ grains are interwoven throughout the ceramic matrix, forming a characteristic interlocking microstructure. It is noteworthy that distinct pores with relatively large sizes can be observed in SPS-7. The presence of these pores is consistent with the decrease in relative density shown in Figure 3B, suggesting that excessive grain growth and volatilization during sintering may have hindered complete densification of the ceramic.

**Figure 5:**
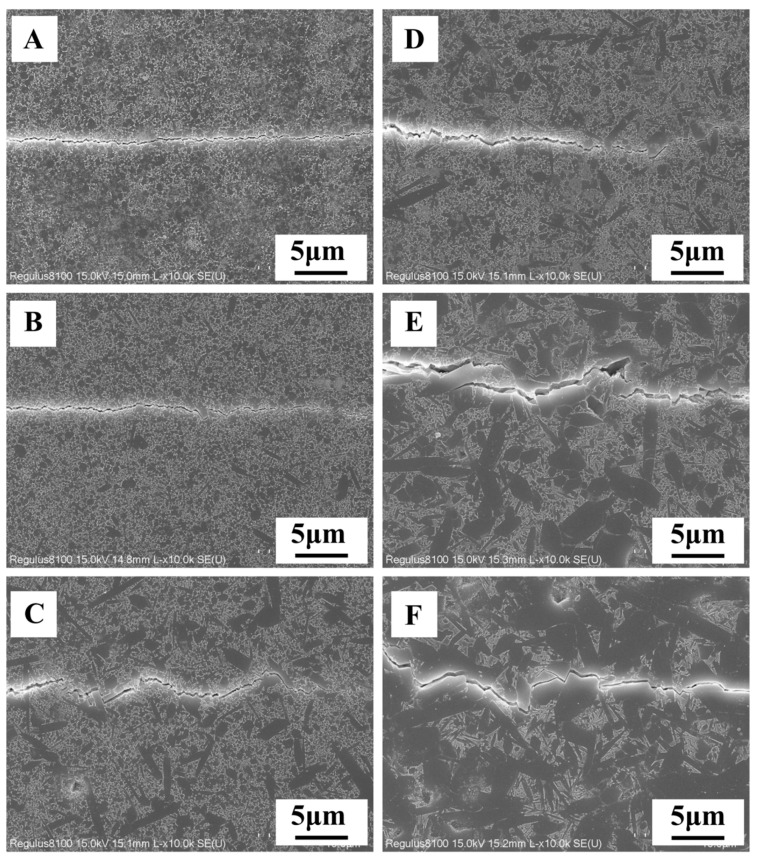
The cracks in SEM images of the as-polished and plasma-etched surfaces of Si_3_N_4_ ceramics: (**A**) HP-5, (**B**) HP-6, (**C**) HP-7, (**D**) SPS-5, (**E**) SPS-6, (**F**) SPS-7.

Figure 5 shows the crack propagation behavior around the Vickers indentation impressions on the polished surfaces of the samples. Significant differences in crack propagation patterns can be observed among the various specimens. In HP-5, the cracks tend to propagate along relatively straight paths. This behavior can be attributed to the fine and uniformly distributed α-Si_3_N_4_ grains, which provide limited resistance to crack propagation. As a result, the cracks can readily traverse the microstructure without undergoing significant deflection. Similarly, the cracks in HP-6 also exhibit nearly linear propagation paths. Although a small amount of β-Si_3_N_4_ is present, the elongated grains remain relatively small and are more susceptible to transgranular fracture, making them ineffective in promoting crack deflection. A distinct change in crack propagation behavior is observed in HP-7. The crack paths become noticeably tortuous due to the increased fraction of elongated β-Si_3_N_4_ grains. These rod-like grains promote crack deflection along grain boundaries, thereby increasing the crack propagation path and consuming additional fracture energy. Meanwhile, transgranular fracture of some β-Si_3_N_4_ grains can also be observed. The fracture behavior of SPS-5 is similar to that of HP-7. In contrast, SPS-6 and SPS-7 exhibit significantly larger β-Si_3_N_4_ grains, resulting in more pronounced crack deflection and larger deflection angles during intergranular crack propagation. In addition, crack bridging by elongated β-Si_3_N_4_ grains can be clearly observed, where the grains remain intact across the crack surfaces and hinder crack opening [27,28]. Although the grain size increases substantially in these samples, a limited amount of transgranular fracture is still observed, indicating that crack propagation occurs through a combination of intergranular and transgranular fracture mechanisms [14,29]. The coexistence of these two fracture modes contributes to the enhanced fracture resistance of the Si_3_N_4_ ceramics.

**Figure 6:**
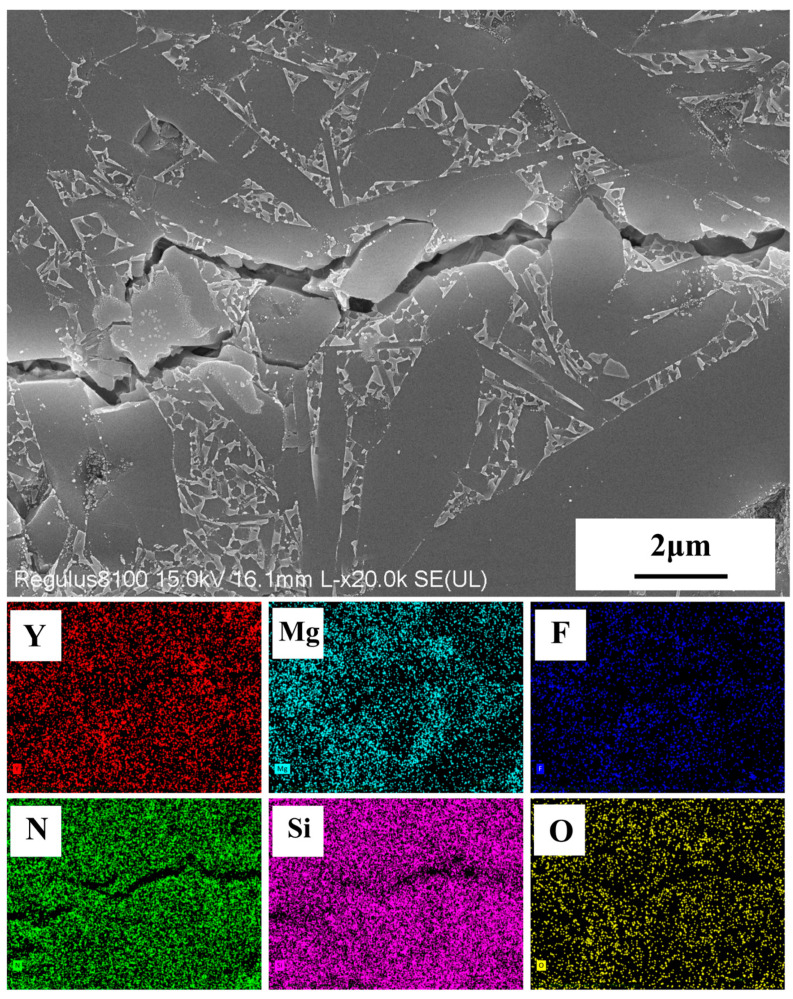
The polished surface micrograph and EDS analysis of the SPS-6 sample.

Figure 6 shows the polished surface micrograph and EDS analysis of the SPS-6 sample. As can be seen, the intergranular phase contains Si, N, Mg, O, Y, and F, whereas the Si_3_N_4_ grains are primarily composed of Si and N. The enrichment of Mg, O, Y, and F within the grain-boundary regions can be attributed to the sintering additives introduced during processing. The phase transformation and grain growth of Si_3_N_4_ ceramics are generally governed by a dissolution–precipitation mechanism. During sintering, the additives react with the native SiO_2_ layer on the surface of the Si_3_N_4_ particles, forming a low-melting liquid phase. Fine α-Si_3_N_4_ particles dissolve into this liquid phase and subsequently precipitate on β-Si_3_N_4_ nuclei, resulting in grain growth and the progressive α→β phase transformation [29,30]. Due to the rapid heating and cooling characteristics of SPS, the liquid phase may not have sufficient time to crystallize during cooling. Consequently, a portion of the grain-boundary phase is retained in an amorphous or glassy state after sintering. Compared with the Si_3_N_4_ grains, this amorphous grain-boundary phase generally exhibits lower strength and stiffness, leading to a relatively weak grain-boundary interface. As a result, crack propagation is more likely to occur along grain boundaries, which is consistent with the intergranular fracture behavior observed in Figure 5 [31,32,33].

**Figure 7:**
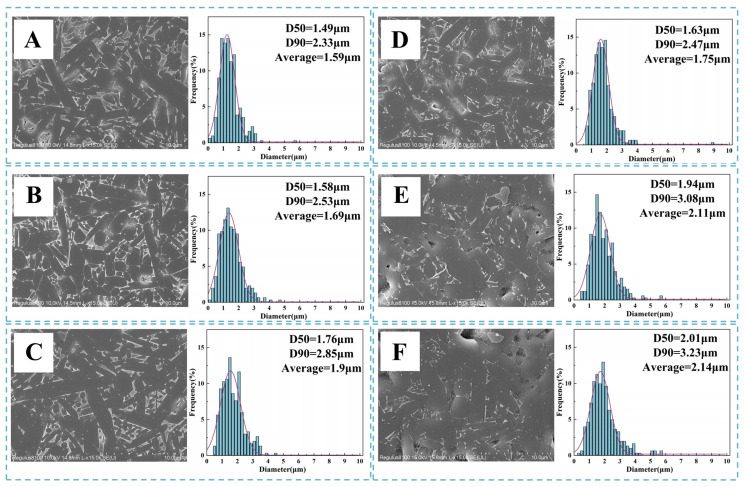
The SEM micrographs of the polished surfaces and grain size of the Si_3_N_4_ ceramic: (**A**) HP-5a, (**B**) HP-6a, (**C**) HP-7a, (**D**) SPS-5a, (**E**) SPS-6a, (**F**) SPS-7a.

Figure 7 presents SEM micrographs of the polished surfaces of the annealed samples together with the corresponding grain size distributions. It can be observed that all samples consist entirely of β-Si_3_N_4_ after annealing, and a significant increase in grain size is evident compared with the as-sintered state. The statistical results show that the average grain diameter of all samples exceeds 1.5 μm. Among them, HP-5a exhibits the smallest grain size, with an average diameter of 1.59 μm, a D50 value of 1.49 μm, and a D90 value of 2.33 μm. In contrast, SPS-7a shows the largest grains, with an average diameter of 2.14 μm, a D50 value of 2.01 μm, and a D90 value of 3.23 μm. The relatively small grain size observed in HP-5a can be attributed to the high fraction of residual α-Si_3_N_4_ and the comparatively low degree of densification in the corresponding as-sintered sample. During annealing, the remaining α-Si_3_N_4_ must first undergo the α→β phase transformation before participating in further grain growth. As the sintering temperature increases from HP-5 to HP-7, the initial β-Si_3_N_4_ content increases, providing more nuclei for grain growth and resulting in progressively larger β-Si_3_N_4_ grains after annealing. A similar trend is observed for the SPS samples. The smaller grain size of SPS-5a compared with HP-7a is also associated with differences in the initial β-Si_3_N_4_ fraction prior to annealing. For SPS-6a and SPS-7a, a significant increase in grain size is observed, accompanied by the appearance of distinct pores within the microstructure. Since the β-Si_3_N_4_ content in SPS-6 and SPS-7 already exceeded 90% before annealing, a highly interconnected network of elongated β-Si_3_N_4_ grains had formed throughout the ceramic. This interlocking grain framework likely restricted further microstructural rearrangement during annealing. Consequently, pores generated by volatilization of the grain-boundary phase were more difficult to eliminate through mass transport and densification processes, resulting in a more pronounced reduction in density compared with the other samples. These results indicate that the initial phase composition and microstructural state prior to annealing play a crucial role in determining the subsequent grain growth behavior and densification characteristics of the Si_3_N_4_ ceramics.

### 3.3. Thermal Conductivity and Mechanical Property

**Figure 8:**
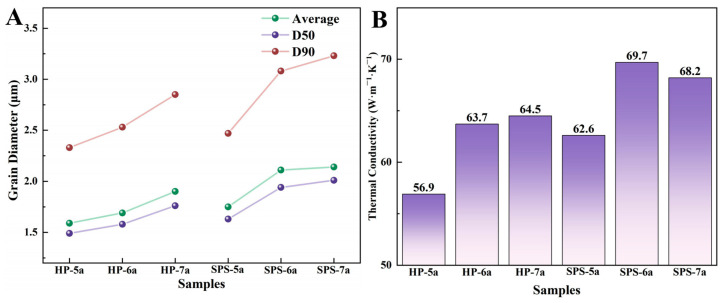
(**A**) The variation in grain size; (**B**) thermal conductivity of Si_3_N_4_ ceramic.

Figure 8A illustrates the variation in grain size, while Figure 8B presents the thermal conductivity of the annealed samples. It can be observed that the trend in thermal conductivity closely follows that of grain size. Among all samples, HP-5a exhibits the smallest grain size and the lowest thermal conductivity, with a value of 56.9 W·m^−1^·K^−1^. As the grain size increases, the thermal conductivity gradually improves. This result is consistent with previous studies, which have demonstrated that grain size is one of the key factors governing the thermal conductivity of Si_3_N_4_ ceramics. The increase in thermal conductivity with grain growth can be attributed to the reduction in grain-boundary density. Since phonon transport is the dominant heat-transfer mechanism in Si_3_N_4_ ceramics, grain boundaries act as scattering centers for phonons. Therefore, larger grains reduce phonon scattering and facilitate heat transport, leading to enhanced thermal conductivity [34,35,36].

However, an anomalous behavior is observed for SPS-7a. Although SPS-7a exhibits a larger average grain size than SPS-6a, its thermal conductivity is lower. This result indicates that grain size is not the sole factor controlling thermal conductivity in the present system. As discussed previously, SPS-7a experienced a more pronounced decrease in relative density during annealing, resulting in the formation of a larger amount of residual porosity. These pores serve as strong phonon-scattering centers and significantly impede heat transport. Consequently, the detrimental effect of porosity outweighs the beneficial effect of grain growth, leading to a reduction in thermal conductivity. As a result of the balance between grain growth and densification, SPS-6a exhibits the highest thermal conductivity among all samples, reaching 69.7 W·m^−1^·K^−1^. These results suggest that achieving high thermal conductivity in Si_3_N_4_ ceramics requires not only the development of large β-Si_3_N_4_ grains but also the maintenance of a high relative density.

**Figure 9:**
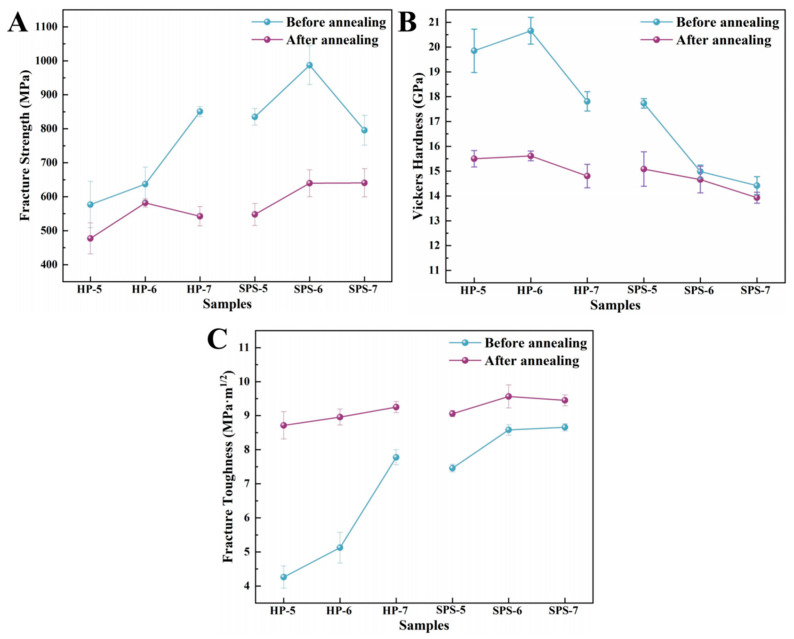
Mechanical properties of Si_3_N_4_ samples: (**A**) flexural strength, (**B**) Vickers hardness, (**C**) fracture toughness.

Figure 9 summarizes the mechanical properties of the samples, including flexural strength (Figure 9A), Vickers hardness (Figure 9B), and fracture toughness (Figure 9C).

As shown in Figure 9A, HP-5 and HP-6 exhibit relatively low flexural strengths, with HP-5 showing the lowest value of 577 ± 68 MPa. This behavior is associated with the high α-Si_3_N_4_ content in these samples. In HP-7 and SPS-5, the β-Si_3_N_4_ fraction increases significantly, accompanied by an improvement in flexural strength. The elongated β-Si_3_N_4_ grains act as reinforcing elements within the ceramic matrix and enhance fracture resistance through crack deflection, crack bridging, crack pinning, and grain pull-out mechanisms, thereby increasing the energy required for crack propagation. In SPS-6, β-Si_3_N_4_ becomes the dominant phase, and the extensive interlocking network formed by elongated β-Si_3_N_4_ grains results in a substantial increase in flexural strength. Although SPS-7 also contains a high fraction of β-Si_3_N_4_, the presence of large pores within the microstructure provides preferential sites for crack initiation, leading to a noticeable reduction in flexural strength compared with SPS-6. After annealing at 1850 °C for 6 h, all samples were completely transformed into β-Si_3_N_4_. However, the relative density decreased while the grain size increased. As a result, all annealed samples exhibited lower flexural strengths than their as-sintered counterparts. This result indicates that the detrimental effect of density reduction outweighs the strengthening effect associated with grain growth and complete phase transformation.

Figure 9B presents the Vickers hardness values of the samples. HP-5 and HP-6 exhibit relatively high hardness, with HP-6 showing the highest value of 20.7 ± 0.5 GPa. Although HP-5 contains a higher fraction of α-Si_3_N_4_, its densification is incomplete, and small residual pores are observed around the α-Si_3_N_4_ grains, which likely accounts for its lower hardness compared with HP-6. As the sintering temperature increases to 1700 °C, the fraction and size of elongated β-Si_3_N_4_ grains increase. Since these grains are considerably larger than the equiaxed α-Si_3_N_4_ particles, the hardness decreases accordingly. A similar trend is observed in the SPS samples. The hardness of SPS-5 is lower than that of HP-5 and HP-6 and continuously decreases as the SPS temperature increases from 1500 to 1700 °C. SPS-7 exhibits the lowest hardness among the as-sintered samples, with a value of 14.4 ± 0.4 GPa. Following annealing, the complete α→β transformation and significant grain growth further reduced the hardness of all samples. SPS-7a exhibits the lowest hardness value of 13.9 ± 0.2 GPa, which can be attributed to its largest grain size and lowest relative density.

As shown in Figure 9C, HP-5 exhibits the lowest fracture toughness, reaching only 4.3 ± 0.3 MPa·m^1/2^. Combined with the crack propagation behavior shown in Figure 5, the fine equiaxed α-Si_3_N_4_ grains are unable to effectively induce crack deflection or contribute to crack bridging and grain pull-out mechanisms. Consequently, cracks can propagate through the ceramic with relatively low energy consumption. A similar explanation applies to HP-6, where the low content and small size of β-Si_3_N_4_ grains provide limited resistance to crack propagation. In contrast, the fracture toughness of HP-7 increases significantly to 7.8 ± 0.2 MPa·m^1/2^. SPS-5 exhibits a comparable toughness value, while SPS-6 and SPS-7 show further improvements, reaching 8.36 ± 0.40 MPa·m^1/2^ and 8.47 ± 0.20 MPa·m^1/2^, respectively. These results demonstrate that the fraction of elongated β-Si_3_N_4_ grains is a dominant factor governing the fracture toughness of Si_3_N_4_ ceramics. The interlocking β-Si_3_N_4_ grains promote crack deflection, crack bridging, and grain pull-out, thereby increasing the energy required for fracture. After annealing, all samples consisted entirely of β-Si_3_N_4_, and substantial grain growth occurred. Consequently, the fracture toughness increased further, with all annealed samples exhibiting values above 8.5 MPa·m^1/2^. SPS-6a achieved the highest fracture toughness of 9.56 ± 0.34 MPa·m^1/2^. These results indicate that when β-Si_3_N_4_ becomes the sole crystalline phase, grain size and relative density also play important roles in determining fracture toughness. Specifically, larger elongated β-Si_3_N_4_ grains enhance toughening mechanisms, whereas excessive porosity can partially offset these benefits. Therefore, an optimal balance between grain growth and densification is required to maximize the fracture resistance of Si_3_N_4_ ceramics.

## 4. Conclusions

Si_3_N_4_ ceramics containing a YF_3_-MgSiN_2_ binary sintering additive system were fabricated by HP and SPS, followed by annealing at 1850 °C for 6 h. The effects of sintering route and sintering temperature on phase transformation, microstructural evolution, thermal conductivity, and mechanical properties were systematically investigated. The main conclusions are summarized as follows:

(1) SPS significantly accelerated the α→β phase transformation of Si_3_N_4_ compared with HP. At 1600 °C, the β-Si_3_N_4_ content of SPS samples exceeded 94%, whereas the corresponding HP sample still contained a substantial amount of α-Si_3_N_4_. After annealing, all samples were completely transformed into β-Si_3_N_4_ with high crystallinity. The initial phase composition strongly influenced the subsequent grain growth behavior during annealing. Samples with a higher β-Si_3_N_4_ content before annealing exhibited more pronounced grain growth after annealing.

(2) The thermal conductivity was closely correlated with grain size and relative density. Larger β-Si_3_N_4_ grains reduced grain-boundary phonon scattering and promoted heat transport. However, excessive density reduction during annealing introduced residual porosity, which became a dominant phonon-scattering source. Consequently, SPS-6a exhibited the highest thermal conductivity of 69.7 W·m^−1^·K^−1^ due to its optimal balance between grain growth and densification.

(3) The mechanical properties were governed by both the β-Si_3_N_4_ content and the microstructural characteristics. The formation of elongated β-Si_3_N_4_ grains enhanced crack deflection, crack bridging, and grain pull-out mechanisms, resulting in improved fracture resistance. Nevertheless, excessive porosity deteriorated the flexural strength. After annealing, all samples exhibited increased fracture toughness owing to the complete β-Si_3_N_4_ microstructure and grain growth, while the flexural strength and Vickers hardness decreased because of grain coarsening and density loss. The SPS-7 sample exhibited the flexural strength of 796 ± 44 MPa, the Vickers hardness of 14.4 ± 0.4 GPa, and the fracture toughness of 8.7 ± 0.1 MPa·m^1/2^. After annealing, the mechanical properties of SPS-7a changed noticeably. The flexural strength decreased to 641 ± 41 MPa, and the Vickers hardness decreased to 13.9 ± 0.3 GPa, whereas the fracture toughness increased to 9.5 ± 0.2 MPa·m^1/2^.

(4) Among all investigated samples, SPS-6a achieved the best overall performance, exhibiting a thermal conductivity of 69.7 W·m^−1^·K^−1^ and a fracture toughness of 9.56 ± 0.34 MPa·m^1/2^. The results demonstrate that controlling the phase transformation behavior prior to annealing and maintaining sufficient densification during grain growth are critical for achieving high-performance Si_3_N_4_ ceramics.

The present work provides insight into the sintering and grain-growth behavior of Si_3_N_4_ ceramics containing non-oxide sintering additives and offers guidance for the design of high thermal conductivity and high-toughness Si_3_N_4_ ceramics.

## Figures and Tables

**Table 1:** Composition ratios and sintering route of Si_3_N_4_ ceramic materials.

	Si_3_N_4_	YF_3_	MgSiN_2_	HP		SPS	
	Molar Ratio (%)			Temperature (°C)	Time (min)	Temperature (°C)	Time (min)
HP-5	93	2	5	1500	60		
HP-6				1600	60		
HP-7				1700	60		
SPS-5						1500	5
SPS-6						1600	5
SPS-7						1700	5

## Data Availability

The original contributions presented in this study are included in the article. Further inquiries can be directed to the corresponding authors.

## Abbreviations

The following abbreviations are used in this manuscript:

HP	hot pressing
SPS	spark plasma sintering
GPS	gas-pressure sintering
